# Development and Application of Patient-Derived Cancer Organoidsin Clinical Management of Gastrointestinal Cancer: A State-of-the-Art Review

**DOI:** 10.3389/fonc.2021.716339

**Published:** 2021-10-29

**Authors:** Ruobing Zhang, Tiantian Guo, Lulin Ji, Yirui Yin, Shuitu Feng, Weihong Lu, Fei Zhang, Maoshu Zhu, Shugang Liu, Jinhua Jiang, Fanwei Zeng

**Affiliations:** ^1^ Organoid Research Center, Xiamen Broad Creation Biomedical Institute, Xiamen, China; ^2^ Institute of Neuroscience, School of Medicine, Xiamen University, Xiamen, China; ^3^ Department of General Surgery, Xiamen Branch, Zhongshan Hospital, Fudan University, Xiamen, China; ^4^ Oncology Department, Xiamen Haicang Hospital, Xiamen, China; ^5^ Department of Obstetrics and Gynecology, Xiamen Branch, Zhongshan Hospital, Fudan University, Xiamen, China; ^6^ Central Lab, The Fifth Hospital of Xiamen, Xiamen, China; ^7^ Department of Traditional Chinese Medicine, The Fourth Hospital of Hebei Medical University, Shijiazhuang, China; ^8^ Department of Interventional Oncology, Renji Hospital School of Medicine, Shanghai Jiaotong University, Shanghai, China

**Keywords:** gastrointestinal cancer, patient-derived gastric organoid, gastrointestinal, organoid, cancer

## Abstract

Human gastrointestinal cancer (e.g., gastric cancer and colorectal cancer) has been a leading cause of cancer-related deaths worldwide and has imposed a great threat to the public health. Although early-stage gastrointestinal cancer can be effectively treated by surgery, followed by postoperative chemotherapy, patients with advanced gastrointestinal cancer often exhibit poor prognosis and cancer relapse due to the absence of effective personalized treatment strategies. Patient-derived cancer organoid technology has been rapidly developed in recent years, and its emergence has opened up an unprecedented approach to model human cancers *in vitro*. Patient-derived cancer organoids involve the *ex vivo* culture of fragments of freshly resected human tumors that retain the histological features of original tumors. This review thoroughly discussed the evolutionary process of human gastrointestinal organoids cultured since 2009, and highlighted the potentials of patient-derived cancer organoids in clinical management of gastrointestinal cancer in terms of advances achieved in cancer modelling compared with conventional modelling methods, high-throughput drug screening, and development of personalized treatment selection. Additionally, the current limitations of patient-derived cancer organoids and the potential solutions to overcome these problems were summarized.

## Introduction

Gastrointestinal cancer is one of the most common types of cancer, accounting for 21% of all types of human cancer. It is also one of the leading causes of death, imposing a remarkable threat to the public health worldwide (1, 2) Gastrointestinal cancer generally consists of three types of cancer: gastric cancer (GC), colorectal cancer (CRC), and liver cancer (2). CRC and GC are the two most frequently diagnosed gastrointestinal cancers (3). Meanwhile, CRC is also the third leading cause of cancer-related mortality globally (9.4%) followed by liver cancer (8.3%) and GC (7.7%) (3).There are two distinct types of gastric adenocarcinoma, intestinal (well-differentiated) and diffuse (undifferentiated), which have a distinct morphologic appearance, pathogenesis, and genetic profiles (2). According to the reported statistics, gastrointestinal cancer imposes a huge economic burden on patients, their families, and healthcare systems.

Despite advances achieved in treatment strategies, e.g., extensive resection and the addition of new drugs to chemotherapy regimens, conventional treatment strategies have failed to improve survival for a variety of tumors (4). For instance, according to the Lauren’s classification system, gastric adenocarcinomas can be divided into two major histological types, diffuse type, and intestinal type. The intestinal type is characterized by cohesive cells which form gland-like structures, while for the diffuse type, tumor cells lack cell-to-cell interactions and infiltrate the stroma as a single cell or small subgroups, leading to a population of non-cohesive, scattered tumor cells. Although the Lauren’s classification system can date back to 1965, it is still widely accepted and employed by pathologists and physicians and represents a simple, while robust classification system (5, 6) However, the World Health Organization (WHO) classification recognizes four major histologic patterns of GC: tubular, papillary, mucinous and poorly cohesive (including signet ring cell carcinoma), plus uncommon histologic variants (6). Although histopathological classification systems are extensively applied in clinical settings, they often accompany limitations on making medical decisions (7). Therefore, genetic testing can be employed to determine inherited cancer risk or to obtain a genetic “fingerprint” of a tumor. GC is classified into four subclasses: tumors positive for Epstein-Barr virus (EBV), microsatellite unstable tumors, genomically stable tumors, and tumors with chromosomal instability (6). Sadanandam et al. presented a CRC classification system, associating with cellular phenotype and responses to therapy. They classified CRC into 5 subtypes: stem-like, transit amplifying [TA], enterocyte, goblet-like, and inflammatory type, of which the TA type could be further subdivided into 2 sub-groups based on different responses to epidermal growth factor receptor (EGFR)-targeted therapy (8). However, making genotype-based clinical decisions is associated with some challenges as the gene sequencing often leads to detection of a minimal number of actionable mutations, and they mainly suffer from a lack of clinically approved targeted therapies (9). Therefore, there is an urgent need for preclinical models for developing further effective targeted therapies for gastrointestinal cancer.

The conventional methods that are used for developing personalized cancer models include two-dimensional (2D) culture of cancer cell lines and patient-derived xenografts (PDXs) (10). PDXs precisely recapitulate the molecular properties and biology of the disease, making them effective preclinical tools for assessing anti-cancer drug activities (11, 12). Although the 2D culture systems of cancer cell lines are cost- and time-effective, due to the inherent flaws of traditional 2D culture, it fails to correctly imitate the architecture and microenvironments *in vivo*, which makes 2D-cultured cells different from cells growing *in vivo* in terms of morphology, proliferation, cell-cell, and cell-matrix inter-connections, signal transduction, differentiation, etc. (2). PDXs are established by collecting fresh tissue specimens from cancer patients and directly implanting them into immunocompromised mice, and they may represent more realistic preclinical models as they closely resemble the tissue architecture of primary tumors, including interactions between other cell types (e.g., stroma and endothelium) (10). PDX models preserve the histologic appearance of cancer cells and retain intratumoral heterogeneity. However, it often takes 4-8 months to develop a PDX model, which is longer than the expected survival of the majority of patients with GC (13).

Therefore, to fill the gap between *in vitro* cell lines and *in vivo* animal xenografts, a newly developed culture system was termed organoid culture, which is applicable for generating CRC organoids, and it can be used as a promising preclinical model for gastrointestinal cancer. The advent of three-dimensional (3D) culture has been accompanied by rapid advancement in the past few decades, as evidenced by the increasing number of studies in this area, including preclinical drug screening, cancer stem cell maintenance, and differentiation, abnormal signal transduction, etc. (2). PDXs involve the *ex vivo* culture of fragments of freshly resected human tumors that retain the histological features of original tumors and are maintained in the animal-derived extracellular matrix (ECM) with the supply of cancer-specific growth factors for future use (14). In 2009, the term ‘organoid’ has been conferred a somewhat restricted meaning, that is, a self-organizing 3D structure grown from stem cells, mimicking *in vivo* architecture and multi-lineage differentiation of the original tissue in mammals. Organoids can be derived from two types of stem cells: (i) pluripotent stem cells (PSCs) and (ii) organ-specific adult stem cells (ASCs) (15). The potential of organoids to complement existing model systems and extend basic biological research, medical research, and drug discovery into a more physiologically relevant human setting is becoming ever more widely appreciated. However, the development of organoid technology is still in its infancy compared to established cell lines and animal models (14). At present, patient-derived gastrointestinal organoids from both normal and tumor tissues can be rapidly established in a large amount with satisfactory success rates and a relatively comprehensive recapitulation of molecular and morphological characteristics of the original tissue samples (2). Thus, they possess enormous potentials in preclinical cancer modelling and clinical applications for treating gastrointestinal cancer.

This review thoroughly discussed the evolutionary process of human gastrointestinal organoids cultured since 2009, and highlighted the potentials of patient-derived organoids (PDOs) in clinical management of gastrointestinal cancer in terms of advances in cancer modelling compared with conventional modelling methods, high-throughput drug screening (HTS), and development of personalized treatment selection.

## Procedure of Human Gastrointestinal Organoid Culturing

With the establishment and maturation of the organoids, they have possessed the characteristics of the original tissues and are highly advised to study multiple human gastrointestinal diseases (e.g., viral and bacterial infections, as well as various types of cancer).

**Figure 1:**
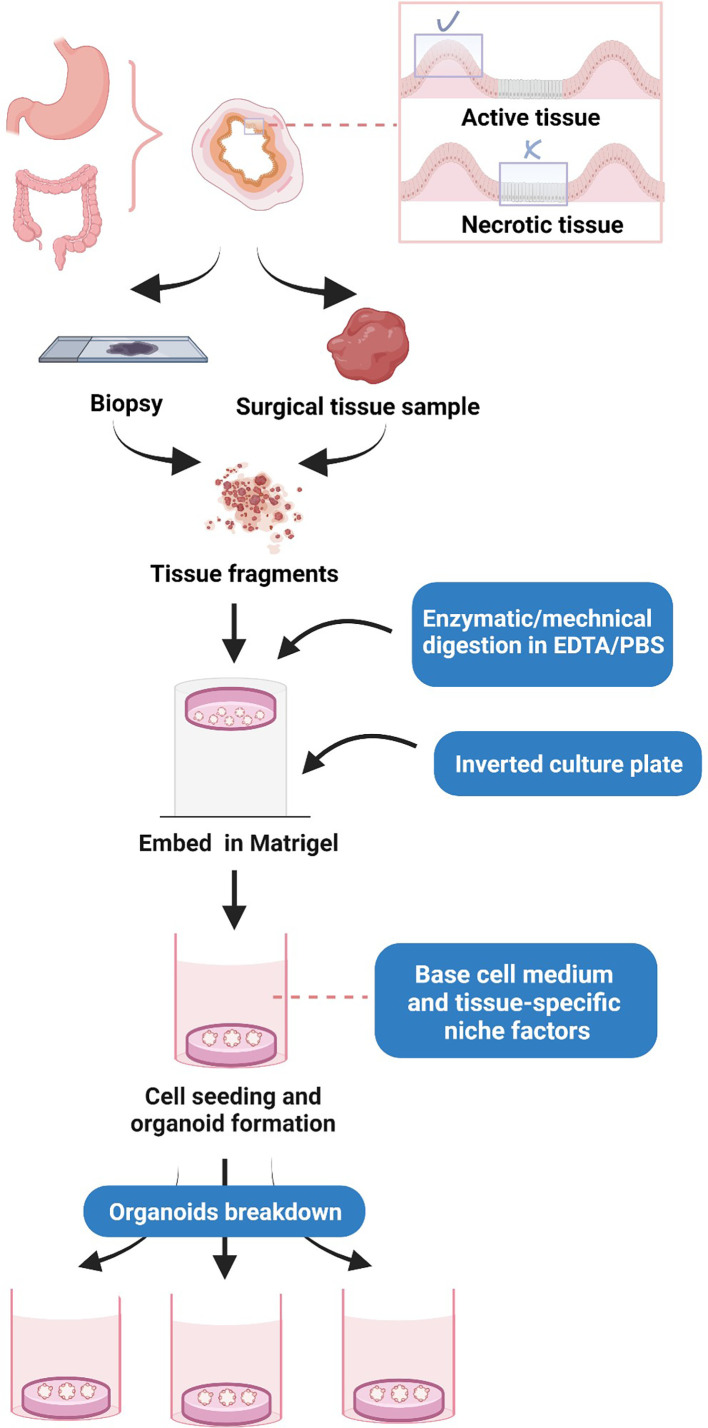
Cultivation of human gastrointestinal organoids.

Before processing, the critical step is to take the areas with more tumor cells, such as those occupied by active tissue, rather than necrotic tissue (16) (**Figure 1**). The processing of human gastrointestinal organoids includes fragmentation and digestion of tissue specimens, cell seeding, and propagation, as well as budding, replication, and several rounds of passaging of organoids (4). In this process, a tissue sample is first washed with ethylenediaminetetraacetic acid (EDTA) buffer, and minced tissues are subjected to enzymatic digestion, typically through different types of collagenases and hyaluronidase, and filtered through a cell filter to produce a single-cell suspension. The cells were cultured in Dulbecco’s modified Eagle’s medium/nutrient Ham’s mixture F-12 (DMEM/F12) (4). The isolated cells are then seeded into Matrigel, a reconstituted basement membrane extract that is rich in laminin, growth factors, entactin/nidogen, type IV collagen, and heparan sulfate proteoglycan (perlecan). Matrigel is a basement membrane ECM, which allows the 3D expansion of organoids (4). A basal culture medium (an DMEM/F-12 supplemented with 1% penicillin/streptomycin, 1% GlutaMAX, and HEPES 10 mM) and tissue-specific adult stem cells are then added to the cultivation after Matrigel polymerization to facilitate cell propagation and organoid formation (4). The cell culture may initially form a spherical cystic structure containing multiple cell types due to the induced differentiation (4). After that, organoids enter into budding and replication stages, followed by passaging, where the produced organoids are digested and resuspended to produce more substantial biological materials for further experiments (4).

## Establishment of *In Vitro* Normal Human Gastrointestinal Organoid Culture System

### Small Intestinal Organoids

In 2009, a number of scholars attempted to describe the establishment of long-term culture conditions under single crypts undergoing multiple crypt fission events, while simultaneously generate villus-like epithelial domains, in which all differentiated cell types are present (**Table 1**). They concluded that intestinal crypt-villus units are self-organizing structures, which can be established from a single stem cell in the absence of a non-epithelial cellular niche (17). They also identified a gene, Lgr5, which is specifically expressed in cycling crypt base columnar (CBC) cells that are interspersed among the Paneth cells (17). A single-crypt unit or an epithelial stem cell with high expression level of Lgr5 exhibited high success rate of organoid formation (17). This Matrigel-based, long-term culture system developed for single crypts could realize *ex vivo* formation and maintenance of the crypt-villus epithelial morphology *via* addition of a series of growth factors promoting cell stemness (17). The activation of Wnt signaling pathway was believed to be crucial for crypt proliferation; therefore, Wnt agonist R-spondin 1 could induce marked crypt hyperplasia *in vivo* (17). Moreover, bone morphogenetic protein (BMP) and epidermal growth factor (EGF) were involved in the culture medium for crypt growth, while noggin was found essential for passaging of organoids (17). Nonetheless, for single cell-derived organoid culture, two additional elements, including Rho kinase inhibitor (Y-27632) suppressing embryonic stem cells and Notch-agonist peptide (Jagged 1) maintaining crypt proliferation, were added into the culture medium to induce organoid formation (17). Besides, 90% of single crypts and 6% of the Lgr5-expressing stem cells were developed and self-organized into 3D intestinal organoids exhibiting an indistinguishable appearance (17).

**Table 1:** Evolution of culture systems for human gastrointestinal organoids.

Study	Organoid type	Niche factors
Sato et al. (17)	Murine small intestine	500 ng/ml R-spondin1, noggin, 10-50 ng/ml EGF, 10 mM Y-27632, 1 mM Jagged 1
Barker et al. (18)	Murine stomach	50% Wnt 3A conditioned media, 100 ng/ml FGF 10, 10 nM gastrin
Sato et al. (19)	Murine colon	1000 ng/ml R-spondin1, noggin, 50 ng/ml EGF, 10 uM Y-27632 (first 2 days), 100 ng/ml Wnt 3A
Sato et al. (19)	Human colon	1000 ng/ml R-spondin1, 100 ng/ml noggin, 50 ng/ml EGF, 10 uM Y-27632 (first 2 days), 100 ng/ml Wnt 3A, 10 nM gastrin, 10 mM nicotinamide, 500 nM A83-01, 10 uM SB202190, 100 ng/ml FGF 10
Bartfeld et al. (20)	Human stomach & Gastric cancer	10% R-spondin1 conditioned medium, 10% noggin conditioned medium, 50 ng/ml EGF, 10 mM Y-27632, 50% Wnt conditioned medium, 1 nM gastrin, 200 ng/ml FGF 10, 2 mM TGFβi (Optional: 10 mM nicotinamide)
Wetering et al. (21)	Human colorectal cancer	20% R-spondin1 conditioned medium, 10% noggin conditioned medium, 50 ng/ml EGF, 10 nM gastrin, 10 mM nicotinamide, 500 nM A83-01, 3 uM SB202190, 10 nM Prostaglandin E2, 10 uM LY27632
Broutier et al. (22)	Murine liver	Isolation: expansion medium + 50% Wnt 3A conditioned medium, 100 ng/ml noggin; Expansion: 10% R-spondin1 conditioned medium, 50 ng/ml EGF, 10 nM gastrin, 10 mM nicotinamide, 100 ng/ml FGF 10, 25 ng/ml HGF; Differentiation: expansion medium – R-spondin, nicotinamide, HGF or FGF 10
	Human liver	Isolation: expansion medium + 30% Wnt 3A conditioned medium, 100 ng/ml noggin, 10 uM Y-27632; Expansion: 10% R-spondin1 conditioned medium, 50 ng/ml EGF, 10 nM gastrin, 10 mM nicotinamide, 100 ng/ml FGF 10, 25 ng/ml HGF, 10 uM forskolin, 500 nM A83-01; Differentiation: expansion medium – R-spondin1, nicotinamide, HGF or FGF 10, + DAPT, dexamethasone, bmp7
Broutier et al. (23)	Human liver cancer	50 ng/ml EGF, 10 nM gastrin, 10 mM nicotinamide, 100 ng/ml FGF 10, 25 ng/ml HGF, 10 uM forskolin, 5 uM A83-0, 3 nM dexamethasone, 10 uM Y27632
Nuciforo et al. (24)	Human hepatocellular carcinoma	50 ng/ml EGF, 10 nM gastrin, 100 ng/ml FGF 10, 5 uM A83-01, 3 nM dexamethasone (optional FGF 19)

### Colonic Organoids

Upon successful development of murine small intestinal organoids, the establishment of colonic organoids was also demonstrated (**Table 1**). The previously developed small intestinal culture system (a basal culture medium with EGF, noggin, R-spondin 1, and ENR) was tested, which showed a significantly lower success rate (range, 1-3%) compared with the small intestine (90%), although the initial expansion of colon epithelium was presented (17, 19). Thus, it was attempted to add Wnt3a to ENR medium (WENR) which subsequently increased the plating efficiency by 10 times (19). The WENR system was then tested in terms of formation of *in vitro* human colon crypts (19). However, the formed crypts were only maintained for 7 days (19). Therefore, gastrin (a peptide hormone that stimulates secretion of gastric acid (HCl) by the parietal cells of the stomach) and nicotinamide (a well-known water soluble sirtuin inhibitor) were added to prolong organoid survival (19). Nicotinamide was found highly essential for maintaining culture expansion beyond the initial 7 days, while gastrin did not exhibit adverse effects on both proliferation and differentiation of organoids (19). However, in the absence of the two small molecule kinase inhibitors, Alk 4/5/7 inhibitor (A83-01) and p38 inhibitor (SB202190), human intestinal organoids underwent growth arrest after 10 to 20 population doublings. In contrast, the replicative capacity in the optimized culture condition was extended at least up to 100 population doublings with addition of the inhibitors (19). Therefore, the final human colonic organoid culture (also known as human intestinal stem cell culture, HISC) consists of a basal medium plus EGF, noggin, R-spondin 1, Wnt3a, gastrin, nicotinamide, Alk inhibitor (A83-01), and p38 inhibitor (SB202190) (19). The HISC medium was also found highly appropriate for the long-term cultivation of *ex vivo* human small intestinal crypts (19). Meanwhile, addition of fibroblast growth factor-10 (FGF-10) into HISC medium enabled the Barrett’s epithelium organoids to form budding structures and significantly prolonged duration of cultivation (19). Additionally, withdrawal of Wnt was required for mature enterocyte differentiation in human colon organoids. However, goblet and enteroendocrine cell differentiation remained blocked. It was revealed that nicotinamide and SB202190 strongly inhibited this differentiation, while withdrawal of the two reagents enabled the organoids to produce mature goblet and enteroendocrine cells (19).

### Gastric Organoids

With the successful cultivation of human colonic organoids, scholars concentrated on the cultivation of human gastric organoids (**Table 1**). In previous research, it was attempted to isolate gastric glands from human gastric corpus tissue, and their growth under different culture conditions was observed. Scholars considered the conditions for mouse gastric epithelium, containing EGF, Noggin, Rspondin1, Wnt, FGF-10, and Gastrin (termed ENRWFG) (20). EGF, noggin, and R-spondin 1 were believed to promote the growth of gastric organoids (18). However, development of human gastric organoids under the same conditions was associated with a low success rate and was difficult to maintain (19). The selection of a series of small molecule inhibitors demonstrated that nicotinamide promoted the initial establishment of human gastric organoids, while limited its lifespan. Therefore, it can be used as an optional factor depending on a research’s requirements (20). Addition of TGFβ increased the lifespan to a maximum of 30 weeks, whereas all other factors had no such effect (20). Moreover, addition of prostaglandin E2 (PGE2) induced growth of large cysts and also prolonged the lifespan of the cultures (20). It was further revealed that withdrawal of EGF, noggin, R-spondin 1, or Wnt ligand attenuated organoid formation, and without FGF-10, gastrin, or TGF-βi, the organoid growth only lasted for 10-20 weeks (20). Therefore, the optimal human gastric organoid culture was the ENRWFG system with the addition of TGF-βi (termed ENRWFG_Ti) (20). The organoids derived from single gastric stem cells could be directed into 4 lineages of human stomach (chief cells, mucous neck cells, enteroendocrine cells, and pit cells), and subsequently self-organized into gland and pit domains (20). Addition of Nicotinamide prevented the pit cell maturation (termed ENRWFGNiTi), while the withdrawal of Wnt ligand from this medium enabled the differentiation of pit cells (20).

### Hepatic Organoids

Meanwhile, the efforts of the establishment of hepatic organoid were also made by numerous of previous studies with a comprehensive protocol published by Broutier et al., in 2016 (**Table 1**) (22). In this study both murine and human liver organoids could be derived from the tissue sections for long-term expansion (22). The progression of the formation of both mouse and human *ex vivo* hepatic organoids consists of three steps: organoid isolation, expansion, as well as differentiation (22, 25, 26). For both adult murine and human, ductal and liver progenitor cells derived from liver tissue sections were firstly seeded in the ECM matrix (Matrigel or Cultrex) and cultured/selected with the isolation medium containing factors Wnt 3A, noggin, R-spondin, nicotinamide, EGF, FGF, HGF (22, 25). The selected cells were then expanded under the medium with the removal of Wnt 3A and noggin (expansion medium) (26). For human liver cell expansion, the addition of forskolin (increasing cAMP level to promote ductal proliferation) and A83-01 (TGFβi) were also added (22). The expanded cells were observed to self-organized into a multi-layered, spherical structure consisting of a single-layered epithelial compartment and a pseudo-stratified embryonic liver bud which could be passaged and maintained for months (22). However, in order to obtain the actual hepatic organoids, the progenitor cells within the organoids were required to differentiate into hepatocytes under the differentiation culture medium (22). The withdrawal of R-spondin and nicotinamide were required for murine hepatocyte differentiation, while the additional removal of forskolin together with the supplement of gamma-secretase inhibitor (for Notch signaling inhibition), bone morphogenic protein (BMP7), and dexamethasone were needed for human liver organoids differentiation (27). Excitingly, the differentiated human hepatic organoids acquired liver functions with the production of albumin, bile acid, and cytochrome activity *in vitro* (27). Moreover, with the orthotopic transplantation of the organoid cells back to the tyrosinemia type I liver disease mouse model, the engrafted cells formed clusters of functional hepatic compartments *in vivo* (22). Beyond the establishment of the traditional, simple liver organoids, recent studies have also made efforts on constructing a more complex vascularized liver organoid model by the co-culturing of IPSC-induced hepatic progenitor cells, human umbilical vein endothelial cells (HUVECs) and mesenchymal stem cells (MSCs) (28).

## Establishment and Optimization of Patient-Derived Gastrointestinal Cancer Organoids

Once the culture systems of human gastrointestinal organoids could be thoroughly explored, application of these systems for the establishment of patient-derived, 3D disease models for various human gastrointestinal cancers have significantly attracted scholars’ attention (**Table 2**).

**Table 2:** Summary of patient-derived gastrointestinal cancer organoids.

Study	Cancer type	Plating efficiency
Xie and Wu, 2016 (21)	CRC	8-fold growth within 25 days
Boehnke et al. (29)	CRC	PDOs formed within 4 days
Schutte et al. (30)	CRC	~60% success rate (35 PDOs and 59 PDXs from 106 patients
Roper et al. (31)	CRC	~100% success rate
Toden et al. (32)	CRC	~40% success rate for Ascl2^-^ cells
		~60% success rate for Ascl2^+^ cells
Vlachogiannis et al. (33)	CRC & gastroesophageal cancer	70% success rate for PDOs from 110 fresh biopsies of 71 patients
Liu et al. (34)	CRC	12415 PDOs from a primary tumor (within 14 days); 12610 PDOs from a metastatic tumor (within 14 days)
Mousavi et al. (35)	Primary and metastatic CRC	100% success rate for 26 patients
Ooft et al. (36)	Metastatic CRC	~63% success rate (40 PDOs out of 60 cultures)
Nanki et al. (37)	GC	Defined culture medium: 54.7% success rate (23 lines from 42 specimens)
		GC enrichment medium: 74.6% success rate (44 lines from 59 specimens)
Yan et al. (38)	GC	>50% success rate (46 PDOs from 34 patients)
Steele et al. (39)	GC	PDOs were established for all the 7 patients within 4-7 days
Wang et al. (40)	GC	PDOs were established for all the 3 patients
Seidlitz et al. (41)	GC	20 PDOs were established for 20 patients
Broutier et al. (42)	Primary liver cancer	10 PDO lines were established from 8 patients diagnosed with HCC and CC
Nuciforo et al. (43)	HCC	10 HCC organoid lines were derived from 8 patients
Li et al. (44)	Primary liver cancer	27 PDO lines were established from all patients for the screening of 129 anti-cancer drug

CRC, colorectal cancer; GC, gastric cancer; PDO, patient-derived organoid; HCC, hepatocellular carcinoma; CC, cholangiocarcinoma.

### CRC Organoids

The human CRC organoids that were initially established by Sato et al. (19) excluded R-spondin 1 from the HISC medium since hyperactivation of Wnt pathway was frequently observed in CRC patients (over 90% of cases) (19). Noggin and EGF were found dispensable in the majority of CRC organoids although their withdrawal might decelerate the growth of CRC organoids in a number of cases (19). In 2015, Wetering et al. explored the application of organoids to routinely establish and phenotypically annotate ‘paired organoids’ derived from adjacent tumors and healthy epithelium from CRC patients (25). The healthy colonic samples were cultured under the previously established HISC medium, while their paired tumor organoids were cultivated under the HISC medium with Wnt withdrawal (25). Besides, 22 CRC organoids were generated from 27 tumor samples with a success rate of ~90% (25). The generated CRC organoids successfully recapitulated the heterogeneity, as well as patient-specific phenotypes of primary tumors (25). Pan et al. demonstrated that CRC tumor organoids could develop *in vitro* independent of elements of the Wnt signaling pathway. However, the other factors remained in HISC medium might be essential for the maintenance of cancer stem cells (27). With the establishment of CRC organoid culture system, PDO has been frequently utilized as a novel preclinical tool for the development of personalized cancer therapy.

Several studies employed the same organoid culture medium presented by Wetering et al. (25) with minimal adjustments, including optional addition of FGF-10, PGE-2, or Rho kinase inhibitor (Y-27632) depending on different requirements for organoids. A previous study employed Matrigel, a culture medium [as same as that proposed by Wetering et al. (25)], and additional factors to compose a 3D culture system (21). They successfully constructed a CRC organoid model that grew robustly over 25 days and demonstrated that 2000 cells/well in 96-well plate were a prime seeding density for cells to form organoids (21). Another study described the establishment of an automated platform in 384-well format for 3D organoid cultures derived from CRC patients. The results demonstrated the feasibility of using patient-derived tumor samples for high-throughput assays and their integration as disease-specific models in drug discovery (29). In 2017, a number of scholars reported an integrative pre-clinical approach based on the establishment and extensive molecular characterization of a large CRC biobank consisting of organoids and xenografts derived from a cohort of 106 patients who were representative of all CRC subtypes. Linking molecular profiles with drug sensitivity patterns led to identify novel biomarkers, including a signature outperforming RAS/RAF mutation in predicting sensitivity to the EGFR inhibitor cetuximab (30). Devarasetty et al. described construction of a bioengineered submucosal tissue, or a submucosal organoid, made with primary colonic smooth muscle cells (SMCs) and collagen I (Col I) that included fiber topography similar to *in vivo* ECM. The data were supplemented with image segmentation to analyze and quantify the collagen fibers in organoids. Their results showed that CRC cells (HCT-116) in the aligned condition exhibited decreased cellular proliferation and reduced sensitivity to 5-fluorouracil chemotherapeutic treatment (45).

From 2018 onwards, the tumoroid technology has been extensively applied in human gastrointestinal cancer research. Roper et al. described some detailed protocols to rapidly and efficiently induce site-directed tumors in the distal colon of mice that were based on colonoscopy-guided mucosal injection. Those protocols were employed to deliver viral vectors carrying Cre recombinase, CRISPR-Cas9 components, CRISPR-engineered mouse tumor organoids, or human cancer organoids to mice to model the adenoma-carcinoma-metastasis sequence of tumor progression (31). Previous research assessed the anti-tumorigenic characteristics of oligomeric proanthocyanidins (OPCs) in CRC using a series of *in vitro* models, followed by validation of their findings in an animal model, which was finally validated in tumor organoids derived from CRC patients. The anti-tumorigenic effects of OPCs were confirmed using multiple xenograft experiments that recapitulated their protective effects using patient-derived CRC organoids (32). Vlachogiannis et al. reported a living biobank of PDOs from metastatic, heavily-pretreated colorectal and gastroesophageal cancer patients recruited in phase I/II clinical trials. Their findings suggested that PDOs could be exploited for functional genomics to simulate cancer behavior *ex vivo*, and integrate molecular pathology in the decision-making process of early phase clinical trials (33). Other studies used ASCL2-responsive minigene labelling and matrix-assisted laser desorption/ionization mass spectrometry imaging (MALDI-MSI) to explore the cell composition and spatial distribution of irinotecan in patient-derived CRC organoids, respectively (34, 46). These two studies also adapted the same organoid cultivation system as previously described by Sato et al. (19) and Wetering et al. (25), which reported satisfactory plating efficiency (~60%) within a short period of time (34, 46). In addition to PDOs, a previous experiment established CRC tissue-originated spheroid lines from the PDXs for the high-throughput sequencing of 2427 compounds with a success rate of 100% from frozen-stock PDXs (30/30) (47).

Later, studies mainly concentrated on utilization of PDOs to predict the outcomes of various cancer treatments, in addition to exploration of the molecular mechanism underlying tumor progression. In 2019, a number of scholars found that KRAS mutation in parental CRC cells promoted the growth of corresponding PDOs at both primary and metastatic stages *in vitro* (35). Another study used PDOs as prognostic tools for chemotherapy in treating metastatic CRC patients and reported a success rate of 63% (36). This study demonstrated that the CRC PDO testing of irinotecan predicted the responses of the original tumor specimens from over 80% patients without misclassification (36). Meanwhile, PDO-associated innovations emerged in 2019. A previous study established a “mini-ring” approach for culturing PDOs in lieu of the conventional drop-seeding methods, which premixed patient-derived cell suspension with clod Matrigel and seeded the cells around the rim of the wells of 96-well plate (48). This method could establish organoid lines within 3 days and enable performing HTS within 5 days after seeding, while it avoided organoid transfer during the whole process (48). Another system was proposed for co-cultivation of PDO-T cells, as well as growth monitoring of PDOs (49, 50). The PDOs of CRC patients was developed and expanded in the HISC-Wnt culture medium for at least 2 months, and then, were tagged by nuclear-enhanced green fluorescent protein (eGFP), which were then transferred into a growth medium containing 2% Matrigel (49, 50). Matrigel maintained fluid at the above-mentioned concentration, thus, it realized the effective cell attachment to the bottom of the plate, in addition to the interaction between T cells and tumor cells (49, 50). This system could further mimic the tumor microenvironment (TME) *in vivo* and provide a novel preclinical model for CRC. With the gradual maturation of PDO technology, the overall success rate for establishment of patient-derived CRC organoids was often reported to be more than 90% (51).

### GC Organoids

In contrast to the popularity of CRC PDOs, GC PDOs have been frequently utilized since 2017. In terms of GC organoids, the previously defined cultivation system for human gastric epithelium (ENRWFG(Ni)Ti) was also found highly appropriate for growth of GC organoids (20). This culture medium consists of a basal culture medium, EGF, noggin, Wnt, FGF-10, gastrin, TGFβi (A83-01), R-spondin 1, Y-27632, and Primocin (20). Previous research adopted this system and demonstrated a plating efficiency of ~50% (51). However, another study published in 2018 indicated that the overgrowth of normal gastric organoids could be often observed that reduced the efficiency of GC organoids. In that research, the compositions of the culture medium were adjusted to form a selection gradient for enrichment of GC organoids (37). First, MDM2 inhibitor (Nutlin-3) was added to the culture for selection of TP53 mutations, followed by the withdrawal of ROCK inhibitor (Y-27632) to select Rho kinase-dysregulated cells (37). TGFβ was then added to the medium upon the withdrawal of Alk inhibitor (A83-01) for selecting TGFβ-insensitive strain (37). With the enrichment of GC organoids, the plating efficiency of PDOs was increased from 54.7% to 74.6% (37). Another innovation presented by previous experiments was the modeling of PDOs using esophagogastroduodenoscopy (EGD) (52, 53).

### Liver Cancer Organoids

The human primary liver cancer organoids were more commonly established in recent years. The previous study published in 2017 established PDOs from eight liver cancer patients diagnosed with HCC, cholangiocarcinoma (CC), and HCC/CC combined tumours (42). PDOs in this study were firstly cultured under the renewed human hepatic organoid isolation medium established by the previously mentioned Broutier et al. group with the removal of R-spondin and noggin, and the addition of dexamethasone for two to three weeks, which were then transferred to the classical human liver organoid expansion medium (23). Nonetheless, another patient-derived liver cancer organoid study published a year later has also done some alterations on HCC tumouroid culturing based on the normal liver organoid establishment (43). In this study, the patient-derived HCC organoids were directly cultured with the adapted human liver organoid isolation medium by withdrawing forskolin, nicotinamide, and HGF, as well as adding FGF19 to the medium to promote HCC proliferation (24). Ten HCC tumoroid lines were derived from eight patients using this method (24). Furthermore, a study done in 2019 utilized the same previously reported human liver organoid isolation medium for the culturing of liver cancer PDOs (44). Twenty-seven liver tumoroid lines were successfully established and screened against 129 anti-tumour drugs (38). It seems that the liver cancer PDOs can be established using both the normal isolation medium and the adapted ones with the removal of several niche factors that might interfere with cancer proliferation. However, various alterations of the isolation medium might be required for different types of liver cancers.

## Patient-Derived Cancer Organoids as Preclinical Models for Gastrointestinal Cancer

### Advances of PDOs in Gastrointestinal Cancer Modelling

One of the major advantages of PDOs in modelling different types of cancer is the accurate recapitulation of both genotypical and phenotypical characteristics of the corresponding tumor samples suggested by preliminary data (4). From the genotypical point of view, PDOs can capture distinct, stage-specific, and genetic profiles of their corresponding tumors with expansion of both tissue-specific stem cells and differentiated lineage cells (27, 53, 54). This characteristic of PDOs promotes in-depth investigation of cancer stem cells and disease progression (27). In contrast to cancer cell lines inducing chromosomal instability after several generations, organoids remain stable and conserve genetic alterations of the original tumors over a great number of generations (4, 39). A previous study detected 6 gastric cancer organoids that conserved a stable transcriptome even after 6 months of cultivation (39). Moreover, regarding time-consuming feature of PDXs, PDO models can be established within 2 weeks for further analysis (i.e., drug screening), which are still less expensive (53). Last but not least, in contrast to the NGS that requires the percentage of tumor cells in a specimen, organoids can be generated and expanded from minimum tissue samples without the requirement of the percentage of tumor cells (33). Meanwhile, the somatic alterations that failed to be detected by gene sequencing might also be identified by targeted therapies developed in PDOs (53).

### Validation of How PDOs Recapitulate the Molecular Features of Parental Tumors

Measurements can be undertaken to verify the consistency between PDOs and their corresponding primary tumors, including histopathological, genotypical, and molecular profiling. Histopathological verification is typically carried out by immunohistochemistry (IHC) and fluorescent labelling of the diagnostic markers of certain types of cancer. The common markers for CRC are human epidermal growth factor receptor-2 (HER2), the kirsten rat sarcoma viral oncogene homolog (KRAS), B-Raf proto-oncogene (BRAF), tumor suppressor gene (P53), Adenomatous polyposis coli (APC), Cytokeratin 7 (CK7), CK70, caudal type homeobox 2 (CDX2), Mucin-2 (MUC2), etc. (23). GCs consist of 4 subtypes, involving microsatellite instability (MSI), Epstein-Barr virus (EBV), chromosomal instability (CIN), and genomic stability (GS) (24). Ring finger protein 43 (RNF43) and AT-rich interactive domain-containing protein 1A (ARID1A) are mainly enriched in MIS-GC.RNF43 interacts with Wnt receptors to inhibit gastric cancer-associated Wnt/β-catennin signaling, thereby inhibiting tumor growth (55),While ARID1A encoded product is an essential component of the SWI/SNF chromatin remodeling complex and cooperates with other regulatory proteins, including DNA polymerase and DNA damage repair proteins, to inhibit the occurrence and development of tumors (56).Phosphatidylinositol-4,5-bisphosphate 3-kinase catalytic subunit alpha (PIK3CA) and tumor protein p53 gene (TP53) are mutated in EBV- and CIN-GC, respectively (38). PIK3CA is the P110 α subunit encoding PI3K, which plays a vital role in tumor cell proliferation, differentiation, transport, and metabolism (57). In addition, TP53 plays an important role in cell cycle arrest, cell senescence, apoptosis, differentiation and metabolism (58). PIK3CA and TP53 were highly expressed in gastric cancer cells, suggesting that these single nucleotide polymorphism (SNP) mutated genes may act as oncogenes in gastric cancer (59). Cadherin 1 (CDH1), Ras homologue family member A (RHOA), and RhoGTPase Activating Protein (ARHGAP) can be found in GS-GC (38). E-Cadherin encoded by CDH1 is a vital molecule of epithelial cell homeostasis and plays an important role in intercellular adhesion (60, 61). While RhoA is a member of small GTPases of the Rho family, which is a molecular switch that cycles between a GTP-bound active form and a GAP inactive form, and RhoA activation plays a crucial role in actin cytoskeletal rearrangement (62). In addition, AGRHGAP is involved in regulating GAP activity of RhoA (62). The loss and abnormal expression of the three genes play an essential role in the progression and invasion of cancer cells. Meanwhile, hematoxylin and eosin (H&E) staining is often applied to compare the morphological characteristics between PDOs and their primary tissue samples. The genotypic profiling can be conducted *via* whole-genome/exome sequencing, the next-generation sequencing (NGS), and RNA sequencing, and their results are typically presented *via* heatmaps exhibiting the most frequent mutations of both PDOs and their corresponding tumors. Molecular profiling includes detection of mutations in the genes encoding EGFR through Illumina-targeted gene sequencing or supervised gene selection (screening PDOs against the commonly mutated genes identified in databases or previous studies for certain type of cancer), mutant allele frequency analysis, and pathway enrichment analysis. Molecular profiling is associated with molecular networks/pathways rather than single genes; therefore, Western blotting might also be included in the analysis.

## Clinical Applications of Gastrointestinal Tumour Organoid Culture

As a cost- and time-effective technology preserving genotypical and phenotypical diversities of the original tissues, an organoid is a significant preclinical model for gastrointestinal cancer, and can be broadly applied in clinical settings. First, gastrointestinal organoids can be used to model the process of carcinogenesis (10). The organoids could induce malignancy *via* CRISPR/Cas9 genome editing, which are then subcloned and expanded to reveal the process of tissue-specific mutation accumulation during carcinogenesis (27). Another crucial application of PDOs is to establish cancer-specific organoid biobanks, consisting of organoid lines derived from different patients for making discrimination between different anti-cancer agents or identification of general efficacy and side effects of a potential drug prior to clinical trials (4, 27, 53). Most importantly, organoids also exhibited a broad spectrum of potential applications in the development of precision medicine in terms of their prognostic and predictive values (27).

### Cancer Organoids in Drug Sensitivity Testing

Upon completion of several rounds of cell passaging, organoids derived from different patients may form a relatively comprehensive molecular and histological landscape of particular cancer (4, 53). Recently, the PDOs-driven drugs have been expanded from neoadjuvant chemotherapies to novel small molecule anti-cancer agents to promote the development of targeted therapies (4).

Organoid biobanks for colorectal and gastric cancers have frequently been established for high-throughput screening. A living CRC organoid biobank was established from the paired tumor and histologically normal CRC tissue samples collected from 20 CRC patients (25). Under the standard HISC medium, normal PDOs were found superior compared with diseased PDOs, which was addressed by the removal of Wnt signaling ligands (Wnt3a and R-spondin 1) in tumor organoids due to the frequent overactivation of Wnt pathway in CRC (27, 63). Expectedly, the chromosomal alterations and mutation patterns of CRC in The Cancer Genome Atlas (TCGA) database and in the primary tissue samples were highly captured by this biobank (25). The high-throughput screening performed on the established colorectal PDO biobank generated highly reproducible, differential responses of each drug among all patients with consideration of reproducible drug activities on the same target (25).

In 2018, the establishment of a human gastric cancer organoid biobank was presented, consisting of 17 normal tissues and 46 tumor organoids derived from 34 patients, covering normal, dysplastic, cancerous, and metastatic stages (40). The normal and short-term/long-term tumor organoids were developed in Matrigel with a standard gastric culture medium developed by Barker et al. (18) and Bartfeld et al. (20), and success rates of 90% and 50% could be finally achieved for the establishment of normal and tumor PDOs, respectively (40). The established biobank encompasses all gastric cancer subtypes, such as MSI, EBV, CIN, and GS (64). The molecular heterogeneity and chromosomal instability of the primary tumors were proved to be stably preserved in their corresponding organoids for a long-time through the methods of transcriptome sequencing and exome sequencing (40). The mentioned PDO biobank was screened against 37 anticancer drugs with reproducible results, and common responses for oxaliplatin, epirubicin, and paclitaxel were identified (40). Additionally, several specific gene-drug associations in gastric cancer PDOs were discovered in other studies, including tumor organoids with ErbB2-amplification exhibiting high sensitivity to lapatinib (65) and AKT1-amplified gastric cancer organoids showing exquisite responses to anti-AKT antibodies (MK-2206 and GSK690693) (52, 66). Nonetheless, the effective drugs identified by organoids have not been clinically confirmed, indicating the necessity of conducting further studies to investigate the authenticity of the results of HTS. Furthermore, the previous study published by Yan et al. in 2018 demonstrated that the established GC PDOs from two patients successfully predicted their clinical responses toward 5-FU/cisplatin therapy post-surgery (38). This outcome directly correlated the PDO model responses to clinical tumour responses and provided the clinical evidence for the prognostic value of PDO models (38).

### Cancer Organoids in Developing Personalized Cancer Treatment Strategies

Patient-derived organoids can be utilized to identify the resistant mechanisms of certain anti-cancer drugs, and to develop further effective treatment regimens (27). In the clinical settings, patients may undergo 8-week chemotherapy, following surgical recovery, in which PDOs can be established for personalized therapy selection during this period (4). Alternatively, matched normal and tumor tissues can be obtained from each patient and used to establish healthy-cancerous paired organoid lines for relatively effective anti-cancer treatments with minimal side effects (27). Additionally, one of the major side effects of the majority of anticancer drugs is the acute hepatic toxicity mediated through cytochrome P450; therefore, *ex vivo* cultivation of normal liver organoids could potentially be used to predict *in vivo* toxicity of potential drugs prior to commencing clinical trials (27, 67). Studies demonstrated that the potential adverse effects on heart and kidney induced by novel antitumor drugs might also be predicted *via* the *ex vivo* organoids (10, 68, 69).

In order to model mechanisms of drug resistance, the PDOs are clinically significant for the modelling of co-expansion and activation of T lymphocytes from healthy donors and tumor organoids from patients, predicting the efficacy and potential toxicity of certain immunotherapies (10). A very recent study published in 2019 revealed that co-culturing of PDOs and cytotoxic T lymphocytes could lead to identify the mechanisms of cibisatamab resistance in CRC (70). Cibisatamab is an immunotherapeutic antibody that recognizes and simultaneously binds to Carcino-Embryonic Antigen (CEA) (70). A number of scholars pointed out that CRC could acquire cibisatamab-resistance *via* gradual loss of CEAs on cancer cells since PDOs with high expression level of CEA exhibited high level of drug sensitivity, while PDOs with low expression level of CEA were found resistant to cibisatamab (70). Moreover, it was revealed that the loss of CEA expression in tumor cells was companied by hyperactivation of Wnt/β-catenin signaling pathway (70).

**Figure 2:**
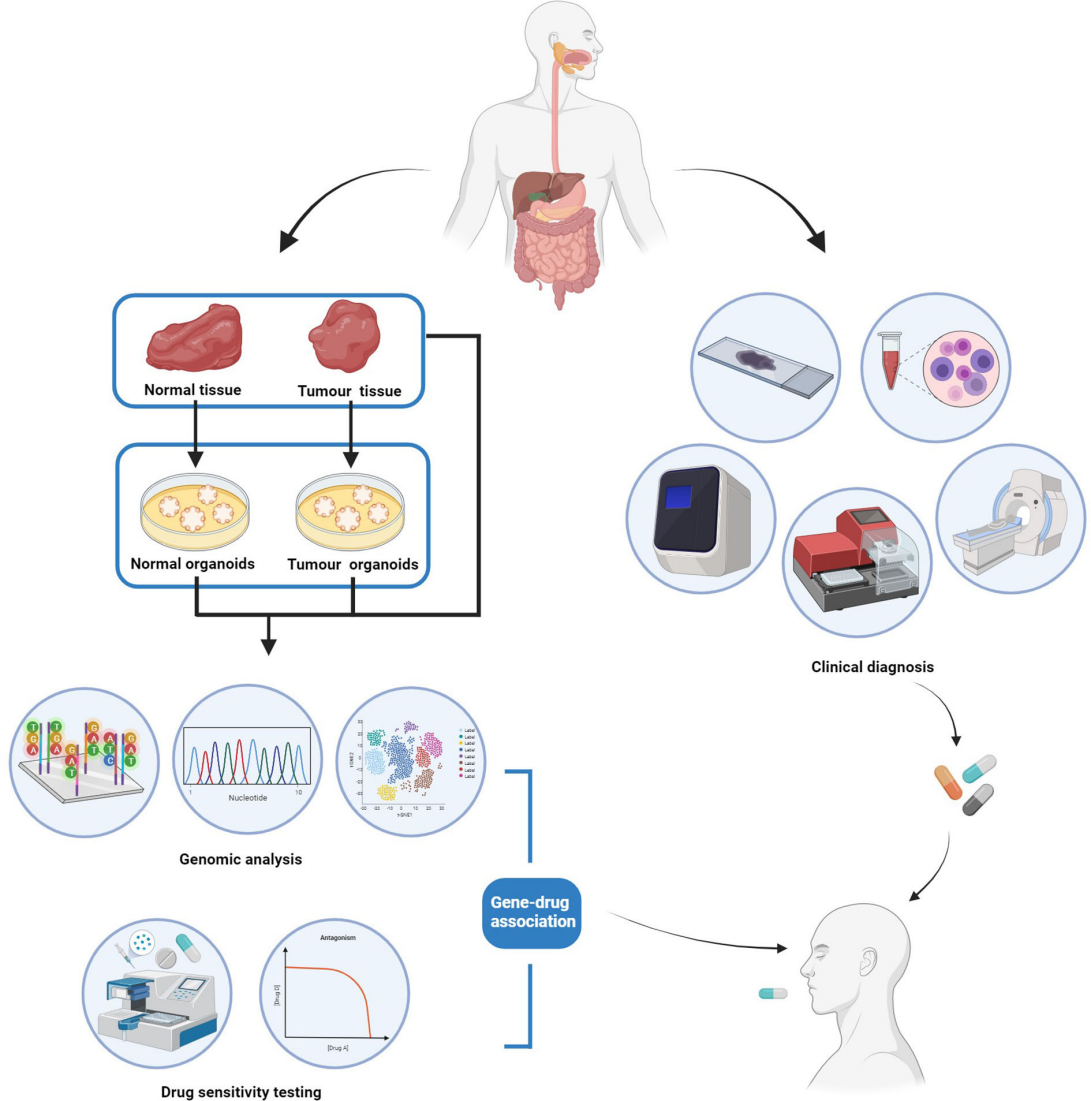
The workflow of patient-derived cancer organoid (PDO) for personalized management of gastrointestinal cancer.

To make personalized therapeutic decisions, patient-derived organoids could be established from both tumor tissues and surrounding normal tissues and then validated by histological staining and genetic profiling of both organoids and their corresponding tissue specimens (**Figure 2**). Once the organoids are validated, drug sensitivity tests can be routinely conducted, and the results of dose-response curves can be presented for each patient under each treatment plan (**Figure 2**). Personalized sensitivity and toxicity of each drug can be further determined *via* its half-maximal inhibitory concentration (IC50) in tumor and normal PDOs, respectively, facilitating the process of making clinical decisions. Moreover, the association between mutation profile and differential drug responses can also be explored for some patients, in order to develop further effective targeted therapies for patients with molecular landscape of gastrointestinal cancer (**Figure 2**).

Recently published studies have mainly concentrated on neoadjuvant chemotherapy. In 2019, a number of scholars thoroughly analyzed the tumor organoids obtained from 4 gastric cancer patients that exhibited diverse molecular and histological characteristics corresponding to their original tumors (41). The established PDO lines were tested against different chemotherapeutic agents (5-fluoruracil (5FU), oxaliplatin, irinotecan, epirubicin, and docetaxel) (41). The results showed that PDOs from one patient was sensitive to both 5FU and epirubicin, while tumor organoids from another patient were fully resistant to the above-mentioned drugs (41). Moreover, it was revealed that patients with HER2 amplification and CDKN2A loss could be effectively targeted by trastuzumab and palbociclib, respectively (41). A later study that obtained cancer organoids from 3 gastric cancer patients indicated that nab-paclitaxel, which is commonly used in the treatment of breast and pancreatic cancers, could also be effective in treating gastric cancer since the nab-paclitaxel demonstrated the lowest IC50 value for the 3 patients in comparison with 5FU and epirubicin (71). Another recently conducted study compared the paired clinical responses and PDO results of CRC patients (36). They found that the PDOs predicted the clinical responses of both irinotecan monotherapy and combined therapy of 5FU + irinotecan with an accuracy of over 80% (i.e., the correlation between clinical responses and PDO results was over 80%) (36). However, this system failed to predict the outcomes of combined therapy of 5FU + oxaliplatin, which might be due to the lack of stromal–immune cell interactions in PDOs (36).

### Clinical Trials Involving in Gastrointestinal Tumor Organoid Culturing

With the maturation of gastrointestinal tumor organoid culturing, several clinical trials have been recently conducted to further examine the feasibility of establishment of tumor organoids from biopsies or to assess the accuracy of PDO-predicted clinical responses of anti-cancer agents.

A previously reported PDO biobank was established from 110 fresh biopsies of 71 metastatic, pre-treated colorectal and gastroesophageal cancer patients who were enrolled in four prospective clinical studies (phase I/II) (33). The success rate of PDO established from biopsies was 70%, which indicated a significant correlation with parental tumor cells (P<0.0001), whereas no correlation was noted with the percentage of necrosis in the tumor tissue (33). A similar clinical study confirmed the feasibility of establishment of PDO from 20 rectal cancer patients. In terms of the implementation of PDOs in clinical management of gastrointestinal cancer, a number of scholars established PDOs from cancer patients to predict patients’ responses to various neoadjuvant and targeted therapies, and facilitates clinical decisions (72–75). In these studies, molecular profiling including genomics and epigenomics were conducted for both original metastatic tissues and patient-derived models (72–75). The drug sensitivity was measured by tumor growth inhibition rate (*in vitro*) and objective tumor response based on standard evaluation criteria like RECIST (*in vivo*), which has demonstrated a high consistency between the predicted and existing datasets for drug sensitivity (72–75).

## Limitation and Prospects of PDOs

Although the patient-derived cancer organoids have shown promising results, there are still some considerations related to implementation of organoid technology in cancer therapy in terms of the authenticity and integrity of the culture system mimicking the actual tumour tissue *in vivo*, as well as the prognostic value of PDOs in clinical decision making.

### PDO Culture Modelling TME Is Required for Comprehensive Recapitulation of Tumour *In Vivo*

The lack of interaction with TME is currently a major drawback for the tumor organoid technology. The importance of crosstalk between cancer cells and their surrounding microenvironments in regulating cancer development (carcinogenesis, tumour progression and metastasis) and treatment response has become highly recognized (76). The complex TME consists of three major components: innate and adaptive immune cellular networks, mesenchymal-derivatives (pericytes and tumour-associated fibroblasts), as well as endothelial vascular networks (76). The immune TME as a crucial component mediating tumorigenesis has been extensively studied for the development of various anti-cancer immunotherapies such as immune checkpoint inhibition, chimeric antigen receptor T cells (CAR-T) therapy, and tumour-infiltrating lymphocytes therapy (77–79). However, the tumour vascular network derived from angiogenesis for oxygen and nutrient supply, as a major cancer hallmark, is often overlooked. A number of scholars concentrated on the integration of epithelial organoid culture with non-epithelial culture, containing stromal, immune, blood, and muscle cells to mimic TME (80, 81). The current PDOs modelling TME can be classified into two subtypes, reconstituted model and native model (76). In the reconstituted TME model, patient-derived tumour cells were encapsulated into Matrigel, the patient-derived peripheral blood mononuclear cells (PBMCs) were then added into the culture medium surrounding the tumour spheroids to imitate tumour TME (76). The native TME models, on the other hand, are patient tumour fragments directly cultured with PDO medium, which preserves the native TME (76).

Furthermore, organoids-on-a-chip combining organ-on-chip technology with organoid has been proposed as an emerging technique to fulfill these requirements.

Organoids-on-a-chip is a precisely engineered cell culture platform to enable *in vitro* cultivation of stem-cell-derived, self-organizing human organs which recapitulate both genotypical and morphological traits of the original tissues (82). This technology achieves this by analysing and identifying the key physiological elements of the target organ including cell composition and physical structure, then constructing a culture platform mimicking the actual physiological microenvironment (82). So far, human lung (the alveolar-capillary unit), multilayered human retina, as well as human non-alcoholic fatty liver disease have been successfully modelled from human-induced pluripotent stem cells or embryonic stem cells (82–84). The advantages of organoids-on-a-chip devices over organoids are that they coculture and compartmentalize different cell types and organize them into their biophysical structure, as well as their ability of emulating the physiological microenvironments such as utilizing cyclic vacuum system to mimic breathing, building channels for vascular perfusion, characterizing diseases by altering the surrounding biochemical components (82–84). Therefore, although this is a novel, unmatured technique at the moment, organ-on-a-chip still holds great potential in medical research for their abilities to recapitulating *in vivo* pathophysiological microenvironments of the original tissue/organ as a precise preclinical model for therapeutic agent development and high-throughput drug screen (82–84).

Meanwhile, patient-derived explants (PDEs) have also been proposed as an alternative for organoids, which can be performed *via ex vivo* cultivation of freshly resected tumor-containing fragments, retaining the intact TME on a specialized collagen gel (85). The histoculture drug response assay of PDEs can be conducted *via* a variety of methods, including submersion method (i.e., completely submerging of tissue fragments in a culture medium), grid method (i.e., keeping a tissue in contact with a medium through a plastic/metal gird-supported matrix, etc. (85–87). PDE culture was soon adapted for the prediction of chemotherapy responses termed as “histoculture drug response assay” with an 86% correct rate in predicting therapy resistance and a 92.1% correlation with clinical drug sensitivity data for gastric and colorectal cancers (88). However, cancerous/stromal tissue ratios and easy contamination should be taken into account in modeling of PDE.

### *Intratumoral* Heterogeneity Compromises the Prognostic Value of PDO Models

Meanwhile, aside from the challenging of modelling TME *in vitro*, the molecular heterogeneity of the parental tumour and its corresponding PDOs also poses an obstacle of utilizing the PDOs to predictive anti-tumour drug effects. A previous study demonstrated that PDOs derived from the same patient with the same single *KRAS* mutation still exhibited intratumoral heterogeneity in the downstream MAPK signalling, which in turn led to different responses to EGFR and MERK inhibitors (89). Therefore, even for PDOs derived from the same patient, different drug screening results might occur during medical decision making. This observation suggests that establishing of subpopulations of PDOs for individual patient is necessary. A more accurate therapy prognosis may be obtained by evaluating the same drug against multiple PDO subpopulations to identify a more comprehensive overall drug response profile for the patient.

In summary, with the maturation of patient-derived gastrointestinal cancer organoid technology for successfully retaining the molecular and histological characteristics of primary tumors, PDO can be used as a promising preclinical platform for both drug screening and development of personalized treatment selection. Although there are still problems in association with tissue-specific matrices, stromal cell contamination, enrichment of certain subpopulations of cancer cells, and the lack of metabolic interactions in TME, resolutions addressing these problems have also been presented such as the development of new matrices, organoid-on-a-chip model, as well as PDE model. Moreover, the emerging implication of the 3D bioprinting technology into organoid construction also has the potential to enable a large-scale production of PDOs for high throughput drug screening. Therefore, PDOs have promising therapeutic potentials for the time-effective, standard, and large-scale production of tumour models that comprehensively recapitulates the *in vivo* circumstances for medical decision making and therapy development. With the ongoing advances made in this field, the PDO technique might also has the capacity to model not only cancer, but also other types of diseases for therapeutic uses.

## Author Contributions

RZ, TG and LJ drafted and revised it and contributed equally to this work. FZe and JJ are the originators and responsible persons of this review; YY, SF, WL, FZh, MZ and SL collected the data, designed the tables and figures. All authors contributed to the article and approved the submitted version. 

## Conflict of Interest

The authors declare that the research was conducted in the absence of any commercial or financial relationships that could be construed as a potential conflict of interest.

## Publisher’s Note

All claims expressed in this article are solely those of the authors and do not necessarily represent those of their affiliated organizations, or those of the publisher, the editors and the reviewers. Any product that may be evaluated in this article, or claim that may be made by its manufacturer, is not guaranteed or endorsed by the publisher.
